# An eternal hunt for glaucoma

**DOI:** 10.1007/s00417-024-06441-w

**Published:** 2024-06-03

**Authors:** Paulus T. V. M. de Jong

**Affiliations:** 1 Department of Retinal Signal Processing, The Netherlands Institute of Neuroscience, Royal Academy of Arts and Sciences, Meibergdreef 47, 1105 BA Amsterdam, The Netherlands; 2 Department of Ophthalmology, Amsterdam University Medical Center, Amsterdam, The Netherlands

**Keywords:** Glaucoma, Primary open angle glaucoma, Angle closure glaucoma, Secondary glaucoma, Historical review, Future research

## Abstract

In the first issue of Graefe’s Archive from 1854, Albrecht von Graefe wrote about glaucoma. Glaucoma comes from the Greek word “glaukos,” gleaming, which was first used by Homer around 800 BCE. Since then, glaukos and glaucoma have taken on many different meanings. The terms blindness, cataract and glaucoma were used interchangeably and twisted together in incomprehensible contexts. Over 2500 years of glaucoma theories were upset by the discovery of the ophthalmoscope in 1851. The first reports of increased intraocular pressure appeared in the mid-seventeenth century, but it took over 200 years for this elevated pressure to be accepted by the ophthalmological community. The discovery of glaucoma simplex in 1861 was an important step forward. What did doctors know about glaucoma before 1850 and why did it take so long to classify glaucoma in its various categories? And why is it that we still do not know what the cause is for primary open angle glaucoma? I will try to answer some of these questions after a historical overview.


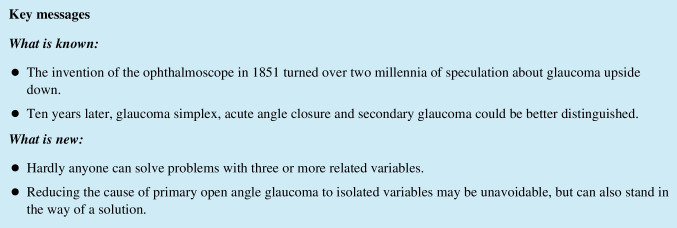


## Introduction

Friedrich Wilhelm Ernst Albrecht von Gräfe, common era (CE) 1828–1870, wrote his thesis on bromide at the age of 19 years under the alias Albertus ab Graefe [1]. Seven years later, he founded the Archiv für Ophthalmologie (AfO), after his death called Graefe’s Archiv, possibly stimulated by his father’s journal [2] or by publications from surrounding countries [3, 4]. The first issue of the AfO was more than half filled with articles by Albrecht von Graefe himself, from then on his pen name, and one was about glaucoma [5]. Because there were more ophthalmological (von) Graefes at that time, I will refer to Albrecht from here on as AvG. AvG was, while publishing his glaucoma article, still in the dark about what glaucoma was. More clarity emerged when Frans Donders and his PhD student Jozef Haffmans introduced in 1861 the concept of glaucoma simplex, what we would now call primary open-angle glaucoma (POAG) [6]. The tangle of POAG, secondary and angle-closure glaucoma (ACG) could then gradually be unraveled.

Between the cornea and the iris is the anterior chamber (AC). For the categorization of glaucoma, it is important whether the circumferential peripheral part of the AC, the anterior chamber angle (ACA) between the iris and the cornea, Fontana’s space in the older literature [7], is open. In the ACA, the aqueous fluid disappears from the eye via Friedrich Schlemm’s canal [8, 9] and an uveoscleral outflow pathway. Broadly speaking, glaucoma is currently defined and subdivided in three types: POAG, ACG and secondary glaucoma. POAG is a retinal and/or nervous disorder of unknown origin; hence, we use the term primary, to hide our ignorance? POAG is a triad, characterized by (a) an open ACA in an externally normal-looking eye without any sign of secondary glaucoma, (b) abnormal thinning of the neuroretinal rim of the optic nerve head, the same as optic disc, which thinning leads to excavation of the disc, and (c) visual field loss (VFL) corresponding with the disc segments with rim loss. The majority of patients with POAG has an elevated intraocular pressure (IOP) [10]. The IOP can vary from 0 to 70 mmHg, is on average 16 mmHg in various populations and its normal range is approximately 8 to 22 mmHg. In ACG, the angle is so narrow or even blocked by the iris, that the aqueous humor cannot drain well out of the eye. Acute ACG usually results in a painfully inflamed, fiery red eye with high IOP up to 60 or 70 mmHg and nausea. A third glaucoma group is called secondary glaucoma, in which the ACA or the drainage system of the eye may be open or blocked due to dozens of demonstrable causes. All these three types of glaucoma can lead to optic nerve-related blindness if left untreated.

In my training, I learned that William Bowman, Donders and AvG, working in the mid-nineteenth century, formed the trio founding modern ophthalmology [11]. Did this imply that doctors were ignorant about ophthalmology or glaucoma before that time? There are numerous good reviews of glaucoma, but I found that only a few authors looked seriously at the abundance of glaucoma literature in the era before 1860. On the 170th anniversary of Graefe’s Archive, I thought it would be appropriate to contribute to our glaucoma knowledge by organizing this historical material in broad terms.

I have opted to divide this article into the following sections. (a) Optic nerve anatomy and optic nerve entry into the eye, the papilla, from 800 before CE (BCE) to 1860 CE. (b) The glaucoma paradigms in approximately the same period. (c) IOP, its measurement and its significance for glaucoma. (d) Visual field (VF) measurements and the introduction of VF examination for glaucoma diagnosis. (e) Comments about treatment will start with historical abuses, followed by the history of iridectomy. Finally, some thoughts on where we might go with our glaucoma research.

## The anatomy of the optic nerve, its entry point into the eye and the optic nerve head excavation from ancient times to the twentieth century

For knowledge about the optic nerve in antiquity, we have to rely on historical giants who not only knew Greek and (neo) Latin well [12–15] but sometimes also Arabic [14, 16] as well as Hebrew [17]. Democritus would have been the first to describe the vitreous in 400 BCE, while the earliest descriptions of the hollow optic nerve and chiasm were found in Aristotle 350 BCE [15]. Galen mentioned the location of the optic nerve at the center back of the eye, as well as multiple openings in this nerve upon its entry into the eye. It is uncertain whether he was referring to the cribrosal plate or to vessel lumina in the optic nerve [15]. Galen is also said to have seen the optic disc in the eye fundus [12] and to consider the retina to be part of the brain [15]. According to Hugo Magnus, by 400 BCE philosophical speculation dominated anatomical knowledge. When later researchers could not find the hollow optic nerves, they suppressed and falsified anatomical knowledge for over a millennium [15].

**Fig. 1:**
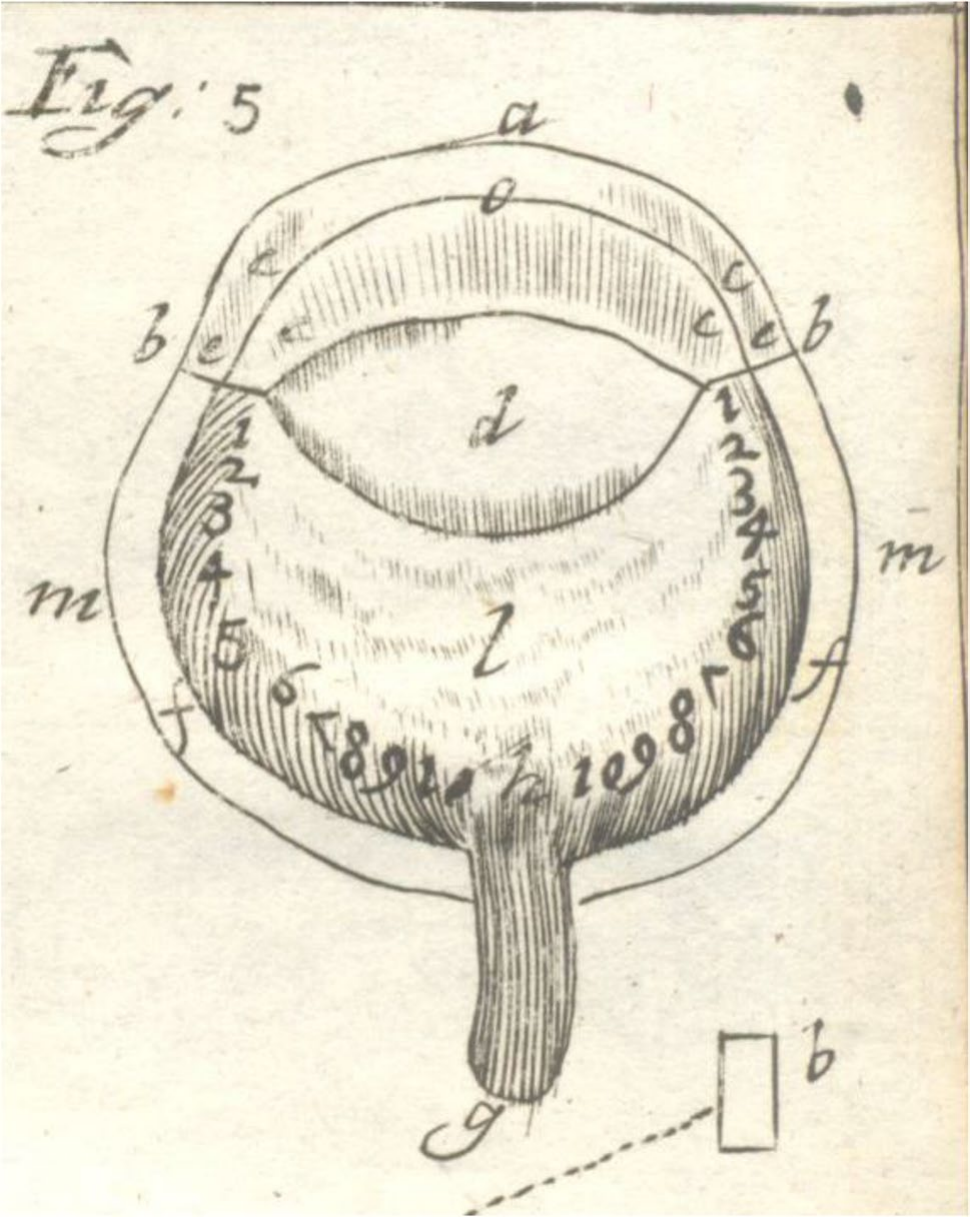
First known description and image of the entry point of the optic nerve into the eye, the papilla, g. by Briggs [18]

**Fig. 2:**
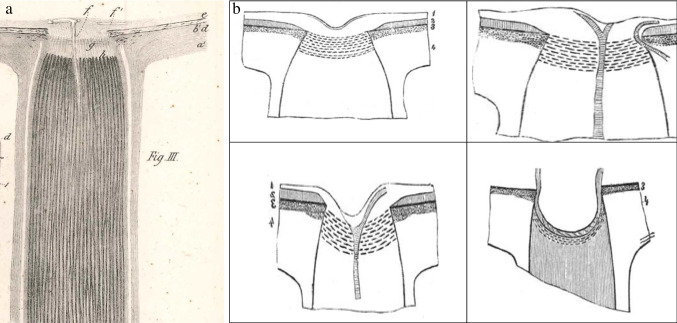
**a** Drawing after a microscopic examination of the papilla by Donders. No dimple is shown in the center of the papilla [24]. **b** Excavations of the optic nerve head and the cribriform plate as drawn by Heinrich Müller [26]

**Fig. 3:**
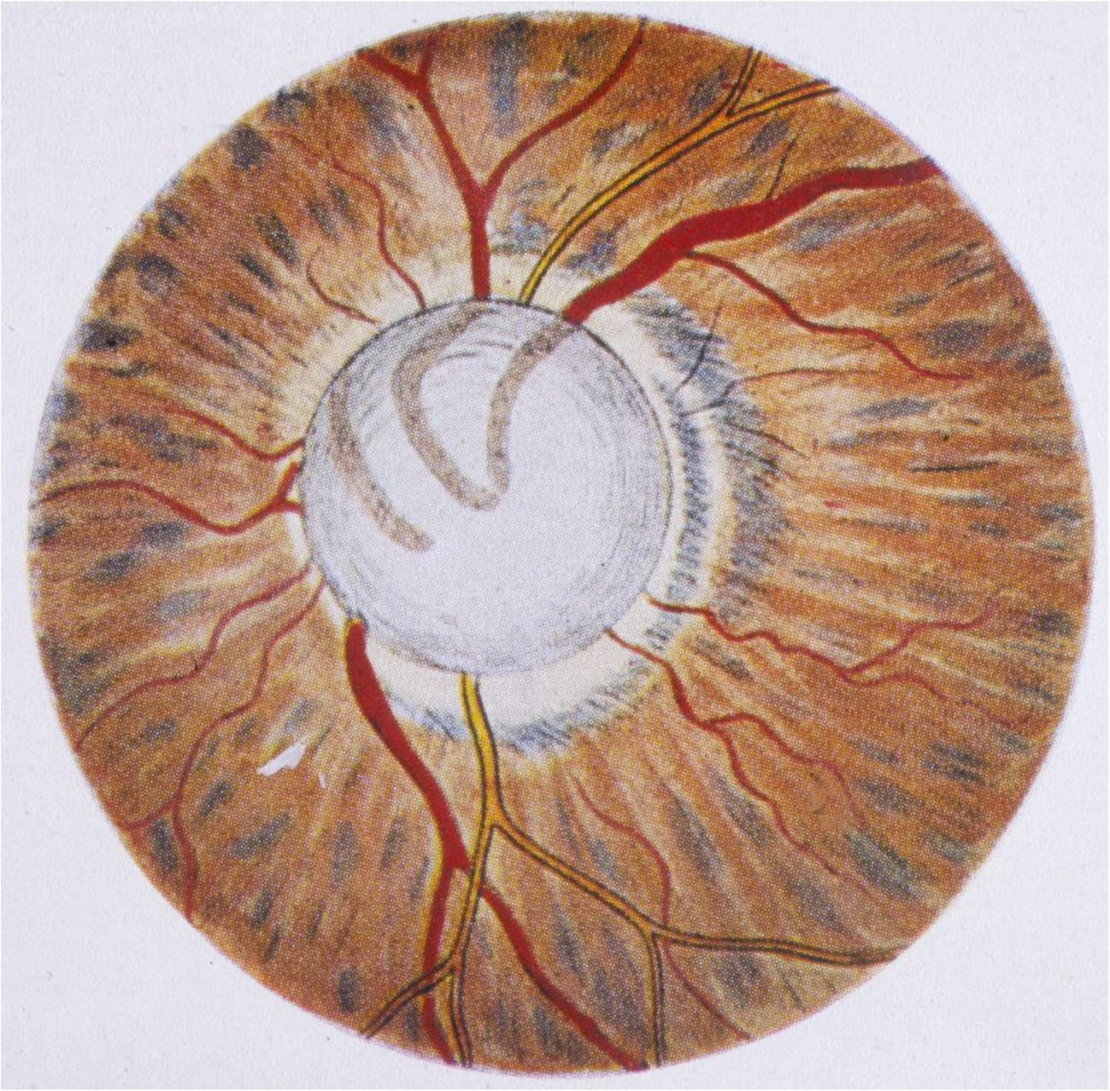
Misinterpreted excavation of the optic nerve head, drawn as a dome, by Eduard Jaeger [27]

One reason for the lack of progress in knowledge of the optic nerve after Galen was the absence of good fixation fluids and magnifiers. This may have led the rediscoverer of the optic nerve that protruded from the eye, William Briggs, to call it in the seventeenth century the papilla, the Latin word for nipple (Fig. 1) [18]. Autopsy reports including clinical data and showing optic damage were collected by Theophile Bonet [19]. In the following years three authors described the cribriform plate [20–22]. The first book on morbid anatomy of the human eye reported on several optic nerve disorders but not on disc excavations or glaucoma [23]. Donders was familiar with fixation techniques of eye tissues, and in 1855 he published drawings of histological sections of the optic nerve and the papilla [24]. He drew the papilla surface as a flat plane without central excavation or cribriform plate (Fig. 2a). Heinrich Müller, dean of the Friedrich Wilhelm Universität in Berlin when AvG wrote his thesis, developed an excellent tissue fixative that was used for many years in histology. In a monograph on the retina, Müller described a crater on the optic nerve head [25]. Soon afterwards, he published several sections through the papilla with variable excavations plus schematically the cribriform plate (Fig. 2b) [26]. Using the newly discovered ophthalmoscope, Eduard Jaeger and AvG described a few years before Müller how a prominent dome developed on the papilla in glaucoma (Fig. 3) [5, 27]. Later, AvG warned against misinterpretation of the papilla shape on ophthalmoscopy, when his student found in only one experimental rabbit eye with ophthalmoscopy and histology that the prominence was an excavation [28]. Next, AvG expressed doubts whether the prominence of the papilla observed by Jaeger and him was not an excavation in reality [5].

## Glaucoma paradigms from 800 BCE to 1850 CE

In ancient times, the word glaukos or glaucoma included hundreds of color shades [29, 30] as well as four types of lens opacities [13, 14, 31]. To spare the reader tedious text, I refer to the end of this article. There you will see an overview of how the meaning of glaucoma, its symptoms and signs have changed over the last 2500 years. The symptoms started to be registered halfway the fourteenth century. Gutta serena, Latin for clear drop, was for hundreds of years a synonym both for blindness without visible abnormalities in the eye and for glaucoma. Lazare Rivière suggested that gutta serena stood for blindness due to optic nerve obstruction through a slimy fluid, creeping from the brain into the nerve [32]. From halfway the fourteenth century more and more signs of glaucoma were noted. It took almost 260 years before it became increasingly accepted that the IOP could rise [110]. Giovanni Morgagni expanded the glaucoma concept by saying that blindness could be due to retinal or optic nerve disease [33]. In the early eighteenth century, Michel Brisseau was the first to state that glaucoma and cataract were two separate diseases; these could be distinguished because glaucoma was located much deeper in the eye [34]. Charles de Saint Yves mentioned that in advanced glaucoma, one can vaguely see objects, only out of the corners of the eyes, where a few undamaged fibers still run [35]. Did de Saint Yves mean lens fibers, despite the fact that Kepler [36] had written 100 years earlier that the lens was not the organ of perception? After all, retinal nerve fibers were unknown at de Saint Yves’ time but lens fibres were [37]. A century after de Saint Yves, Traugott Benedict described “Streifen und weissliche Flecken”, glaucomateuse flecks, in the lens capsule [38] and a little later, Francois Tavignot noted that glaucoma sometimes caused meningitis and death [39]. Doctors were so desperate to find the cause of glaucomatous eye infections, that they put forward explanations in all kinds of physiological or pathological bodily functions. Among others, abdominal plethora [40], pinching of neck vessels by collars or ties [41], arthritis [42, 43], gout [44], menstrual cycle, menopause [45] and hemorrhoids were blamed, leading to strange therapies. Jaeger also brought little clarification, when he classified glaucoma in three types, according to the vascular bed in the eye from which glaucoma developed; from the central retinal, the posterior scleral or the choroidal vessels [42].

I would like to turn the reader’s attention to Jules Sichel, archeologist, entomologist and ophthalmologist. In 1802 CE, he was born into a Jewish family in Frankfurt, Germany. After his doctor’s examination in Berlin in 1823, he converted to the Reformed faith there. Because ophthalmology in Vienna was at a higher level than in Berlin, he left for Jaeger in 1827, where he worked as chef de Clinique for 2 years. Next, he settled in Paris where in 4 years he obtained his French medical degree, graduated in literature from the Sorbonne University and obtained the French nationality. He fluently spoke and read Arabic, French, German, Greek, Hebrew and Latin [17]. In 1836, he opened a clinic for needy and poor eye sufferers. The next year, he wrote a book on cataract [46], and 14 articles on glaucoma followed between 1841 and 1842. The first two provide a general introduction to glaucoma while rejecting several theories about its location in the eye [40, 47]. The next articles contained case reports [48, 49]. Sichel described the results of eye dissections by himself and others and never saw the often described green discoloration of the vitreous. He often found disorganization of the choroid but never mentioned the optic nerve or disc [47]. Was he so fixated on the lens, vitreous and choroid that he forgot about the optic nerve? He considered buphthalmia to be the result of an intrauterine infection [50]. In his articles on the history of glaucoma, significantly more extensive than Jaeger’s [42], he analyzed the texts of over 40 writers, making comments about plagiarism or originality [51–53]. He had nothing good to say about John Taylor [54] and John Woolhouse [55], who made Paris and the continent unsafe with their “charlatan” traits [53]. Amazingly, this highly educated man stuck to his fixed idea that glaucoma was a choroidal disease, despite authors who pointed to the optic nerve or the retina as the cause of visual impairment. Also, Sichel cited nine times an elevated IOP [47, 48, 56] without any comments that elevated IOP could be a player in glaucoma or its visual impairment. Of course, clarity is always easier in retrospect. It should be realized though that these investigators were excellent observers, who had to examine eyes with loupes and often moderate lighting. The ophthalmoscope and the slit lamp did not yet exist, let alone modern developments like the optical coherence tomograph that arrived 150 years later. AvG had a high opinion of Sichel, who only had one problem. “Sein Hauptfehler war – zuviel zu sprechen; mit trägerer Zunge wäre er unendlich liebenswürdiger im Verkehr und weit anerkannter in der Wissenschaft gewesen.” [57] (His main flaw was to speak too much; with a slower tongue he would have been infinitely nicer to deal with and much more respected in science).

At the approach of the first lustrum of the Belgian journal Annals d'Ophtalmologie, the editorial board organized in 1840 CE a prize-winning contest. The questions and issues that needed to be answered could also be asked in 2024. What is glaucoma? What diseases can glaucoma be confused with? Emphasize its differential diagnosis. Disseminate its treatment [58]. Gustav Warnatz revised and improved his prize winning manuscript with the support of the Annals [59]. When he submitted his first version, he was not yet familiar with Sichel's articles. Warnatz quoted Sichel extensively in his revision and may have been influenced by him. Apart from a few expressions such as “Realdefinition” (real definition) of glaucoma and “Amauroseology” (science of blindness), it contained few news. His synonyms for glaucoma, such as amaurosis glaucomatosa, cataracta glaucomatosa, cataracta hyaloidea or cataracta viridis, lacked the terms gutta serena and black cataract, which had been used for hundreds of years.

Let us get back to the main character of the present article. When AvG wrote about glaucoma in the first issue of the AfO in 1854, he also had a quite different idea what glaucoma was compared to the present-day paradigm. The ophthalmoscope hit in 1851 like a bomb [60] and turned the whole glaucoma thinking upside down. AvG started his chapter entitled “Preliminary notes on glaucoma,” as follows. “We see things as the starting point that are only later insignificant consequences. In glaucomatous amaurosis, no constant significant change can be demonstrated in the aqueous, lens, vitreous body, choroid or retina. According to ophthalmoscopy, there are no specific changes in the inner or outer membranes in glaucomatous amaurosis. The only constant findings in this amaurosis are a change in the optic nerve head and a pulsating central retinal artery, spontaneous or after slight digital pressure. Jaeger clearly demonstrated that the optic nerve forms a marked prominent dome with quite variable colors. Even more characteristic was the relation between the vessels and the nerve head. The venules disappear at the bottom of the dome as if cut, in others they can be followed till the center. The peripheral part of the cut vessels is displaced towards the center (Fig. 3). Arterial pulsation is even more characteristic of glaucoma than the optic disc change because the latter is more often seen in amblyopia without other signs of glaucoma. So, we have a clear reason to separate glaucomatous amaurosis due to changes in the central retinal artery from the normal type of glaucoma as a sequence of ciliary vessel disorder.”^1^ [61] AvG examined two post mortem glaucoma eyes but forgot to examine their optic nerve because he was not aware at the time of its significance [61]. That same year he retracted his comment about the prominent papilla, which turned out to be excavated [5]. In 1855, he returned to the pulsatile central retinal artery. “This is constant in glaucoma in the form with subacute choroiditis but often absent in chronic glaucoma where there are no circulatory disorders in the eye. The spontaneous artery pulse does not imply a complete loss of vision, because Donders found that vision disappears when pressing on a healthy eye, shortly after the artery pulse becomes visible. This is an important finding because it provides us with the solution that functional disorders only occur after prolonged, intense pressure” [62]. Next, AvG decided to perform three times paracentesis of the anterior chamber in a man with acute glaucoma, where the IOP was clearly increased. Afterwards the vision improved, and AvG immediately wrote that one cannot draw conclusions about the treatment of this form of glaucoma based on the preliminary findings in one patient [62]. A year later he published on coremorphosis (surgical formation of an artificial pupil) in iritis and iridochoroiditis in a monocular patient. This was a risky procedure in which the iris, which was completely attached to the lens, was pulled off with tweezers to make a hole through which the patient could see again. The next day, the anterior chamber was markedly deeper [99]. I will further describe how AvG came to perform iridectomies and his insights into their effectiveness in the section on therapy.

**Fig. 4:**
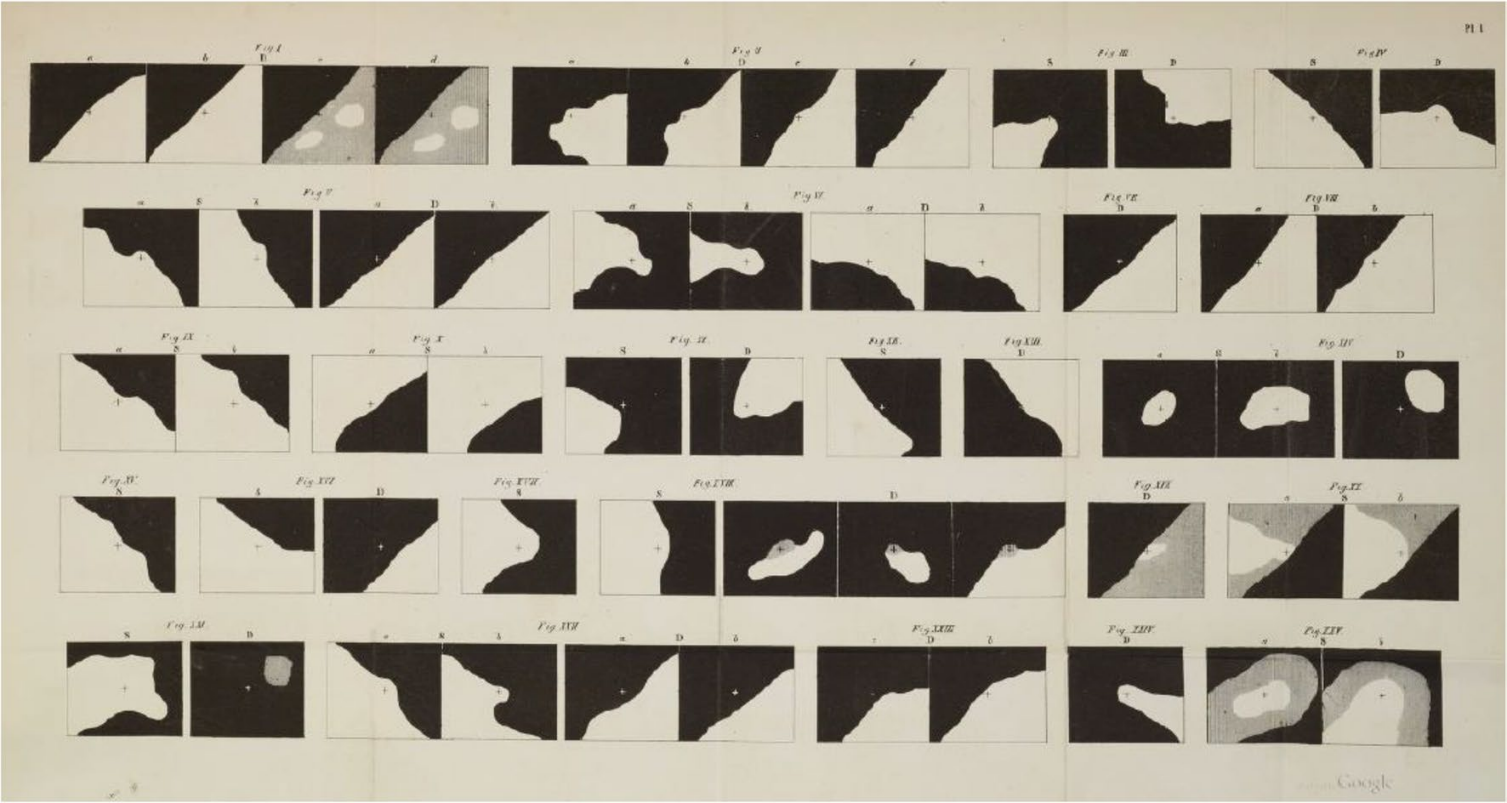
Campimetric visual fields of some 24 patients with glaucoma, attending F.C. Donders clinic, examined by Haffmans [6]

Donders did not agree with the various classifications of glaucoma by AvG and had a PhD student Haffmans study his patient material. He thought that AvG emphasized glaucoma without inflammation not enough. In particular, AvG’s hypothesis bothered him that chronic glaucoma was caused by an increase in IOP and amaurosis with excavation was caused by external pull at the optic trunk. Haffman’s most important conclusions were that there was always an increase in IOP when the disc was excavated, and that there existed both glaucoma simplex without external signs of inflammation and glaucoma with ophthalmitis. Haffmans also stated that the inflammation was the result of glaucoma and not the cause, which he assumed was irritation of nerves to water-producing tissues [6, 63]. He made visual fields on a primitive cardboard campimeter in about 25 patients (Fig. 4) and came to the wrong conclusion that glaucoma caused hyperopia [6].

Schlemm is credited with the discovery of a canal in the ACA in 1827, named after him [8, 9] although Bernard Albinus had described a “red filled circle in the cornea” 50 years earlier [64]. And yet, no one came up with the idea that Schlemm’s canal could be blocked by a bulging iris in case of an occluded pupil or a very shallow ACA. Max Knies found that most fluid left the eye through the pectinate ligament and the basal membranes of the cornea [65]. That same year he showed in rabbits that there was a second drainage to the sclera beyond the equator level, via a thin circular intercellular matrix line in the corneal periphery. In black rabbits the line was pigmented [66]. Knies was so fed up with the standoffish inflammatory and nervous hypersecretion theories on glaucoma, that he decided to conduct clinical and histopathological research on glaucoma eyes [67]. After examining 21 eyes, he came to the important conclusion that both acute and secondary glaucoma were caused by closure of the ACA, in his words space of Fontana [7], and Schlemm’s canal. The local inflammatory infiltrate that he often found around this canal misled him into saying that the inflammation was primary, followed by occlusion of the canal and IOP increase, rather than the other way around [68].

## Intraocular pressure (IOP) and its measurement

**Fig. 5:**
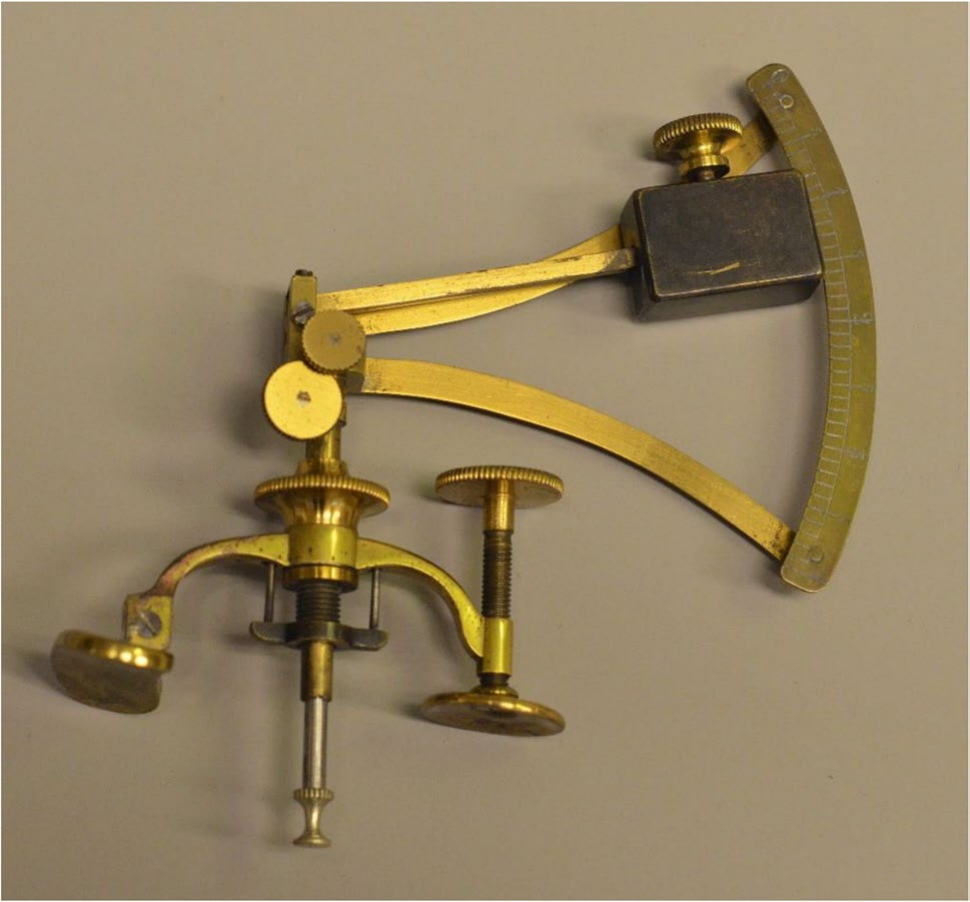
The only tonometer A von Graefe had made. Sent to Donders around 1864 [71–73]. Collection dr. F.P. Fischer foundation, Utrecht University Museum, The Netherlands

**Fig. 6:**
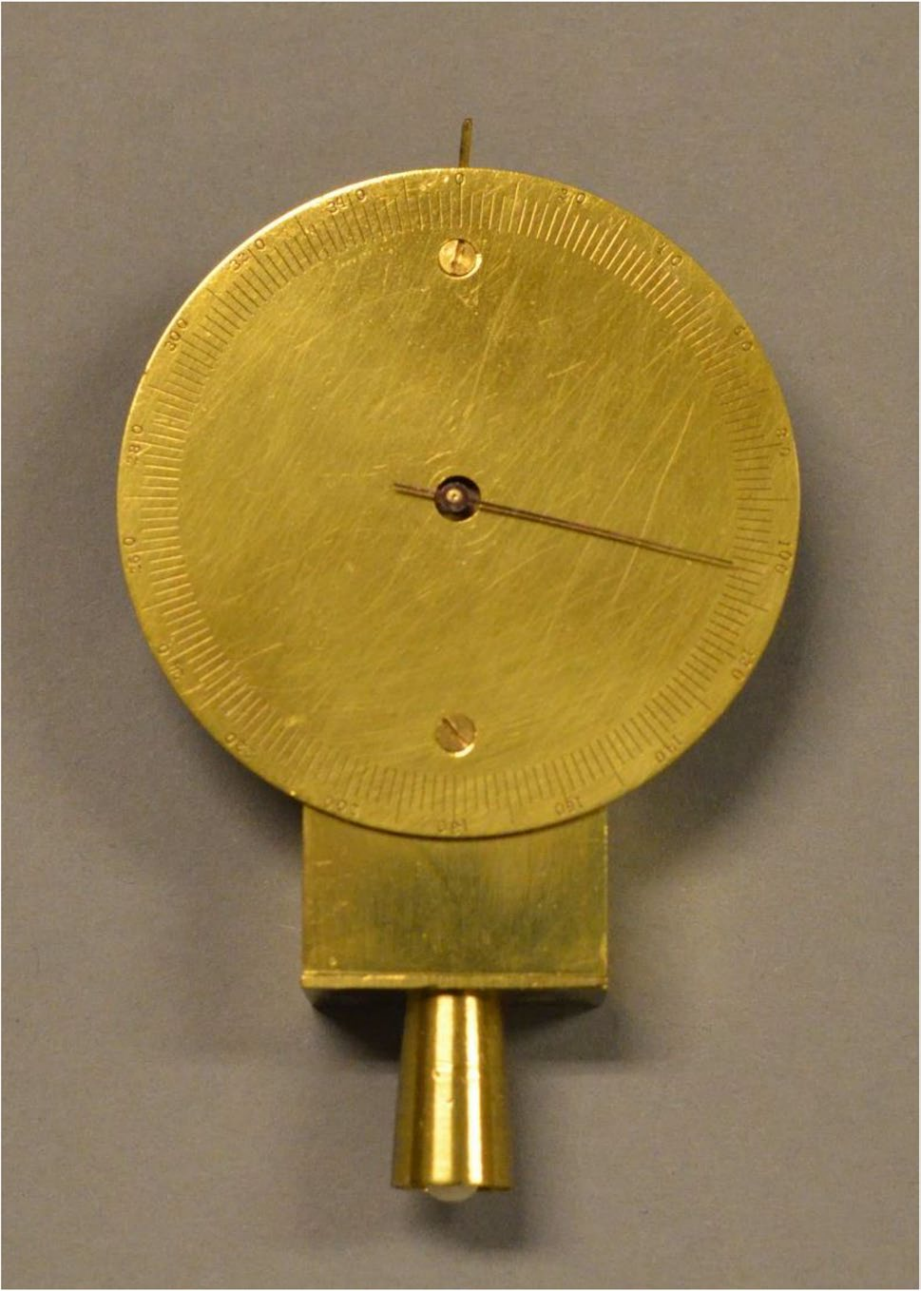
Possibly Donders’ first tonometer from 1863 [78, 79]. Collection dr. F.P. Fischer foundation, Utrecht University Museum, The Netherlands

This seems like a good time to address the IOP. The first explicit comments on the IOP, date from the early-17th century [110]. It took over 200 years for surgeons to understand this better, surgeons, because ophthalmology was at the time seen as their province. All measurements were estimations via palpation with the fingers on the upper eyelid [69], and in this our famous trio AvG, Bowman and Donders encouraged each other again. Bowman recorded the pressure in nine steps from T3 to minus T3, after simplifying his system on advice of Donders [70]. This gave AvG and Donders the idea, independently of each other, of developing a tonometer [71]. ​AvG sent Donders his prototype, which he apparently never published (Fig. 5) [71–73] and regularly wrote Donders to send him his tonometer [74–77]. Donders asked a PhD student to work on the tonometer and Adriaan Monnik quickly came to the conclusion that AvG’s tonometer was unsuitable [72]. Donders presented his own first prototype for which he coined the name ophthalmotonometer (Fig. 6) [78, 79]. He asked various watch and instrument makers at home and abroad, among them Lecoultre in Geneva [80], to improve it on his directions. In total he appears to have designed and had made 11 prototypes, and only the last one had so little friction resistance that it met Donder’s expectations [73, 81]. The essential difference from its predecessors was that it did not measure the amount of transconjunctival indentation of the sclera, but the force required for a standard amount of indentation [72]. Most older readers will have worked with the Hjalmar Schiötz tonometer during their training. Schiötz modified it several times, partly because he initially tested the tonometer on post-mortem eyes. Thereafter, it dominated the top of the indentation tonometers for years [82, 83]. Nowadays, IOP measurement is less important for glaucoma diagnosis than for checking whether IOP-reducing therapy is effective enough. For further applanation tonometer developments, there are excellent reviews [84, 85].

## Visual field (VF) measurement, loss (VFL) and its introduction for glaucoma diagnosis

**Fig. 7:**
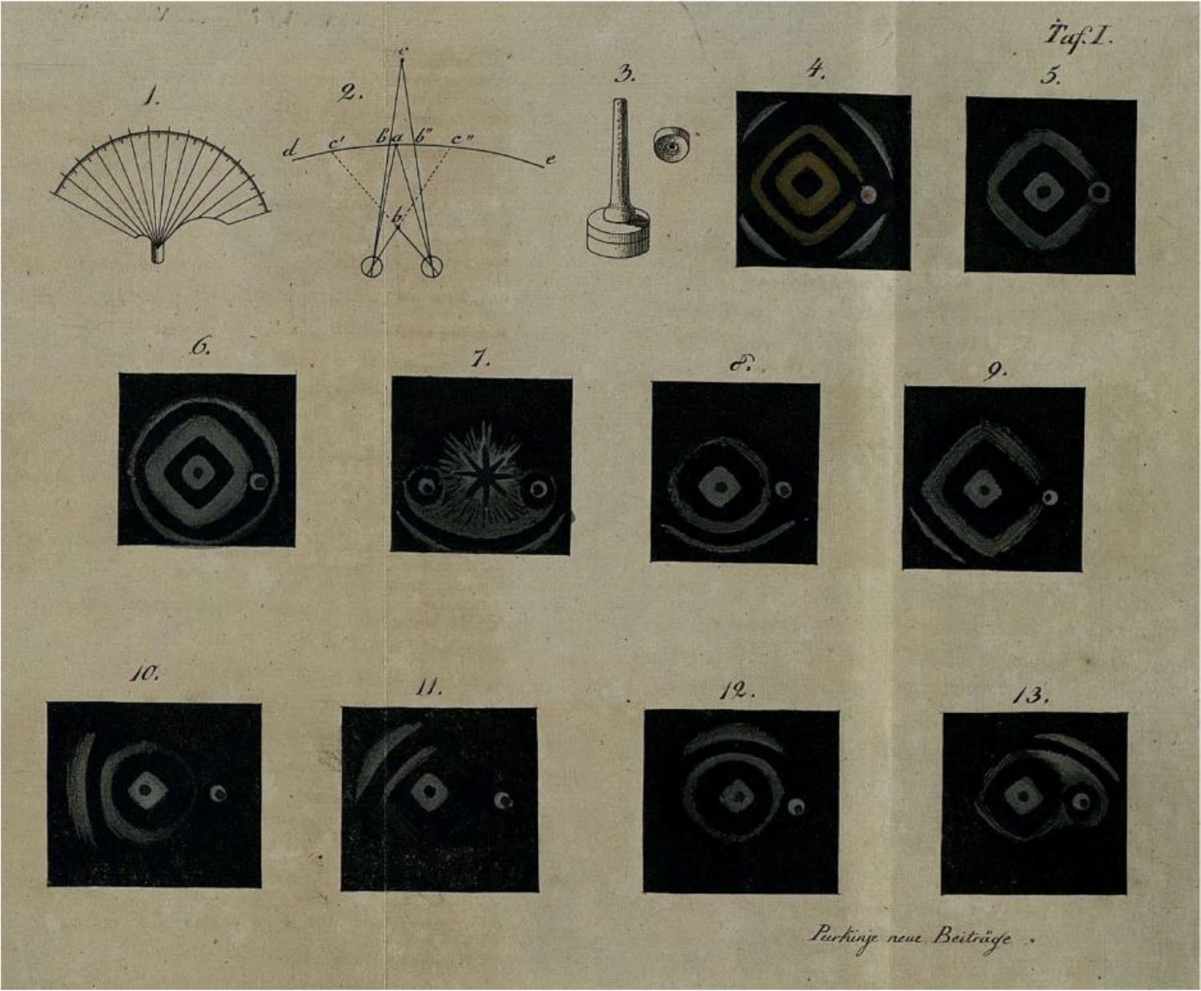
First results of visual field tests, made with a portable campimeter. Purkinje, 1825 CE [90]

**Fig. 8:**
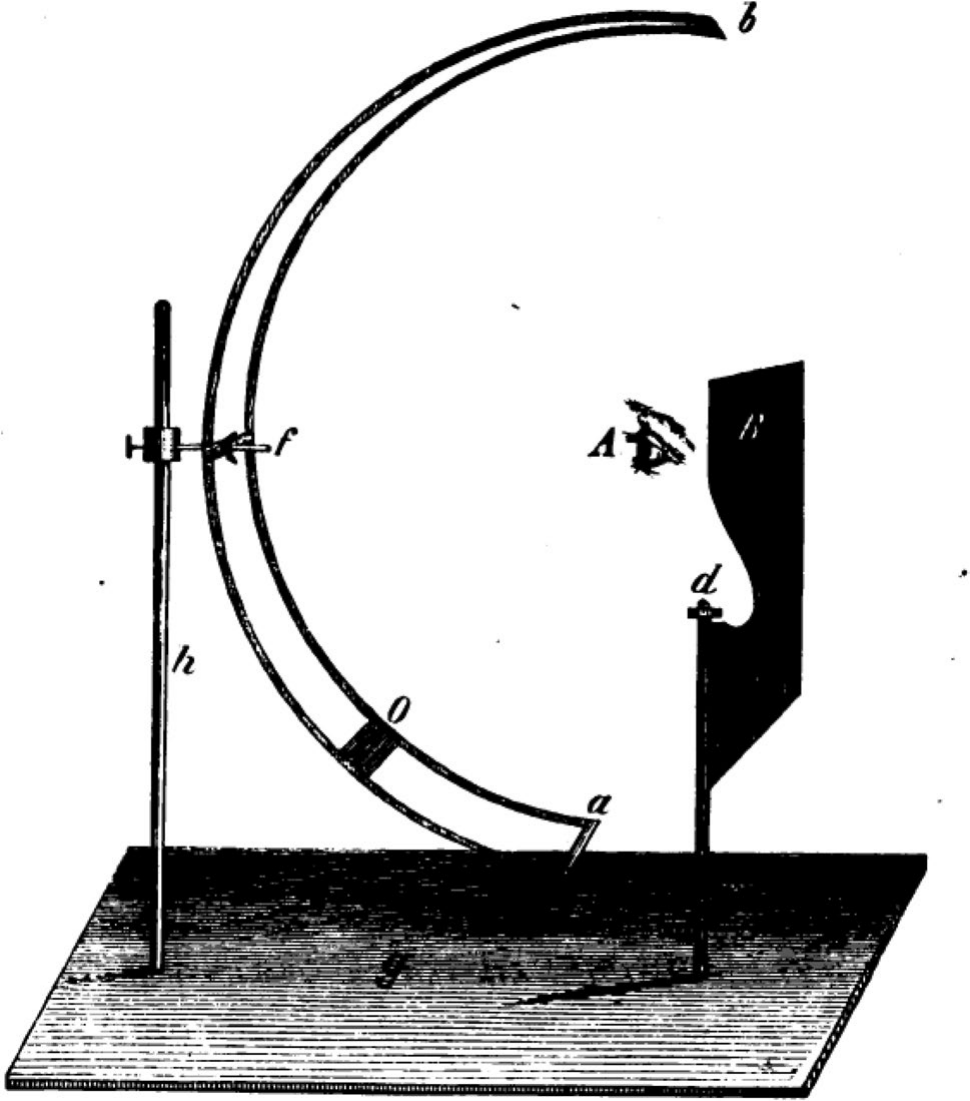
Förster-Aubert arc perimeter from about 1840 CE [92]

The ancients were aware of VFL. Hippocrates knew about hemianopia and Ptolemy calculated 150 CE the extent of a normal VF. He used a primitive perimeter with a calibrated goniometer [14]. Scotoma was the Greek word for dizziness and according to Julius Hirschberg, Sichel first used this word to describe VF defects [86] but Anton Rosas used it earlier [87]. Before Sichel, it was known that the binocular VF, named “campus”, was larger than that of one eye [88] and Thomas Young introduced the concepts of field of view and field of perfect vision [89]. Were they aware that they were possibly repeating Ptolemy? Johann Purkinje used a hand-held campimeter, a piece of cardboard on a stem. He thus found the extent of the monocular VF to be 100 degrees temporally, 80 degrees below and 60 above and nasal to the fixation point (Fig. 7) [90]. A useful comment from AvG was, that VFL is important for diagnosis and prognosis but says nothing about the nature of the disease. He considered VF examination of the highest importance in chronic glaucoma with good visual acuity and a suspect papillary excavation. “As a rule, the dividing line between the affected areas runs diagonally through the field of vision, so that, for example, the outer upper or inner lower part perceives imperfectly [91].” For VF examination one can use a campimeter with a flat surface, a perimeter with a hemisphere, or an arc perimeter with a segment of a sphere. The first improvement on the hand-held campimeter, was the arc perimeter of Aubert-Förster (Fig. 8) [92]. In reviews you can find the developments from manual [93] to automatic perimetry [94] after Haffmans.

## Glaucoma therapies and iridectomies

My cheeks are crimson when I read what our colleagues have done to their patients. I do hope people will not write that about us in 50 years’ time. Out of the countless treatments, one with ice [95], I will pick three, partly to illustrate how distraught the patients with glaucoma often were and how desperate their doctors.

**Fig. 9:**
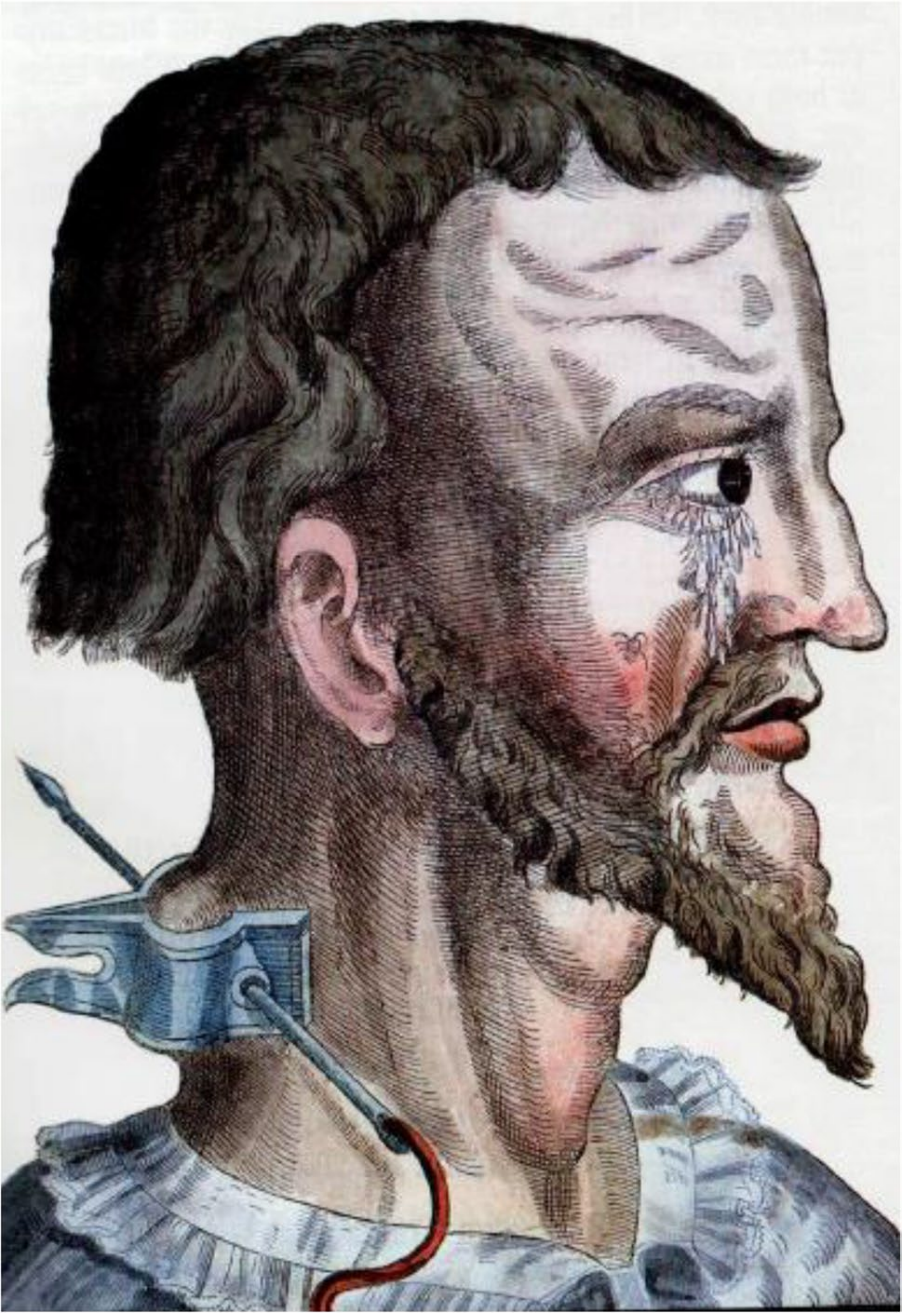
Seton inserted in the neck according to Bartisch, 1583 CE. After piercing the hot nail through the holes in the clamp and the fold of skin, the horsehair was pulled through and cut off. The subsequent festering wound would drain evil juices from the head and eye [96]. Courtesy Kugler Publications, Amsterdam, The Netherlands

George Bartisch, the barber-surgeon from 1583 CE, had curious ideas but also sympathized with his patients. He wrote for example that if one had lived chastely for too long, sperm could rise to the eyes and cause cataract. He fulminated against quacks who couched cataracts in the marketplace and then let people stumble away “like a sow leaving the trough.” Possibly partly to dissuade eye sufferers from suicidal thoughts, he applied a seton, originally made from horsehair, to the neck (Fig. 9). After the red glowing nail was pierced through the skin, the hairs were cut and remained in the skin. Thus, a festering wound was created through which all the evil juices could leave the head and eyes. Bartisch described the symptoms of acute glaucoma, black cataract and gutta serena, but did not use the word glaucoma [96]. Are you not shocked to read that 300 years later, Antonio Scarpa [97] and Sichel [40] were still using linen setons for glaucoma in the neck and around the eyes? And that Hirschberg wrote that he applied setons in his early years and saw them being employed in AvG's clinic? [14]

Louis Gendron suggested draining fluid from the eye in gutta serena, but not how it should be done [41]. William Mackenzie recommended around 1830 CE as treatment for glaucoma, in addition to abstinence from alcohol and smoking, daily belladonna drops. Occasionally also puncturing with a broad knife, at the spot through which couching normally took place. “The instrument should be pushed towards the centre of the vitreous humour, turned a little on its axis, and held for a minute or two in the same position, so that the fluid may be allowed to escape. Removal of the crystalline lens from a glaucomatous eye …..might be advantageous even in the early stage of this disease …. but extraction is an operation, which I would, by no means, venture to recommend for general adoption in such cases.” [98]

The third example is how Sichel who published these articles during AvG’s lifetime, 10 years before the ophthalmoscope arrived, treated his glaucoma patients. He wrote that bloodletting, ointments, eye drops, setons, cauterization, moxas on the mastoid or temple nor thermal baths helped against glaucoma. The doctor had to concentrate on correcting the neuralgic pain, dysmenorrhoea, menopause, and on suppressing hemorrhoids and excess blood in the abdomen [40]. Nevertheless, we read in several of his descriptions mustard plaster and suction cupping on the lower body parts, whether or not after scarification, laxatives [40], 20 leeches near left ear or in neck, seton in neck, leeches around the anus (probably against hemorrhoids) [48], leeches on lower eye lid, blood-letting at feet or arms, moxa’s on temple or mastoid and wheat compresses around feet [48, 49]. Anton Rosas even prescribed leeches in the nose and inside the pubic lips [87]. Sichel warned against operations for cataracta glaucomatosa, but was occasionally persuaded to do so by the patient and family because of severe pain. Vision never got better. He described an operation by Baron de Wenzel Sr on a Hungarian countess. She had a hard, very painful eye, with gutta serena, a dilated, immobile pupil, varicose vessels and a pointed cornea, prior to the development of cataract. The patient, her parents and the local doctor begged him to operate on the cataract and he reluctantly complied. Immediately after opening the eye and removing the lens, bleeding occurred for 10 hours. With a bandage in bed, the countess suffered terrible pain for 6 hours, after which it gradually subsided. After a few days the eye was much softer, the dilated blood vessels had disappeared, the pain attacks were greatly reduced and the eye remained blind. He considered this little consolation and Sichel concluded that a doctor should never recommend such an operation with this result [48].

**Fig. 10:**
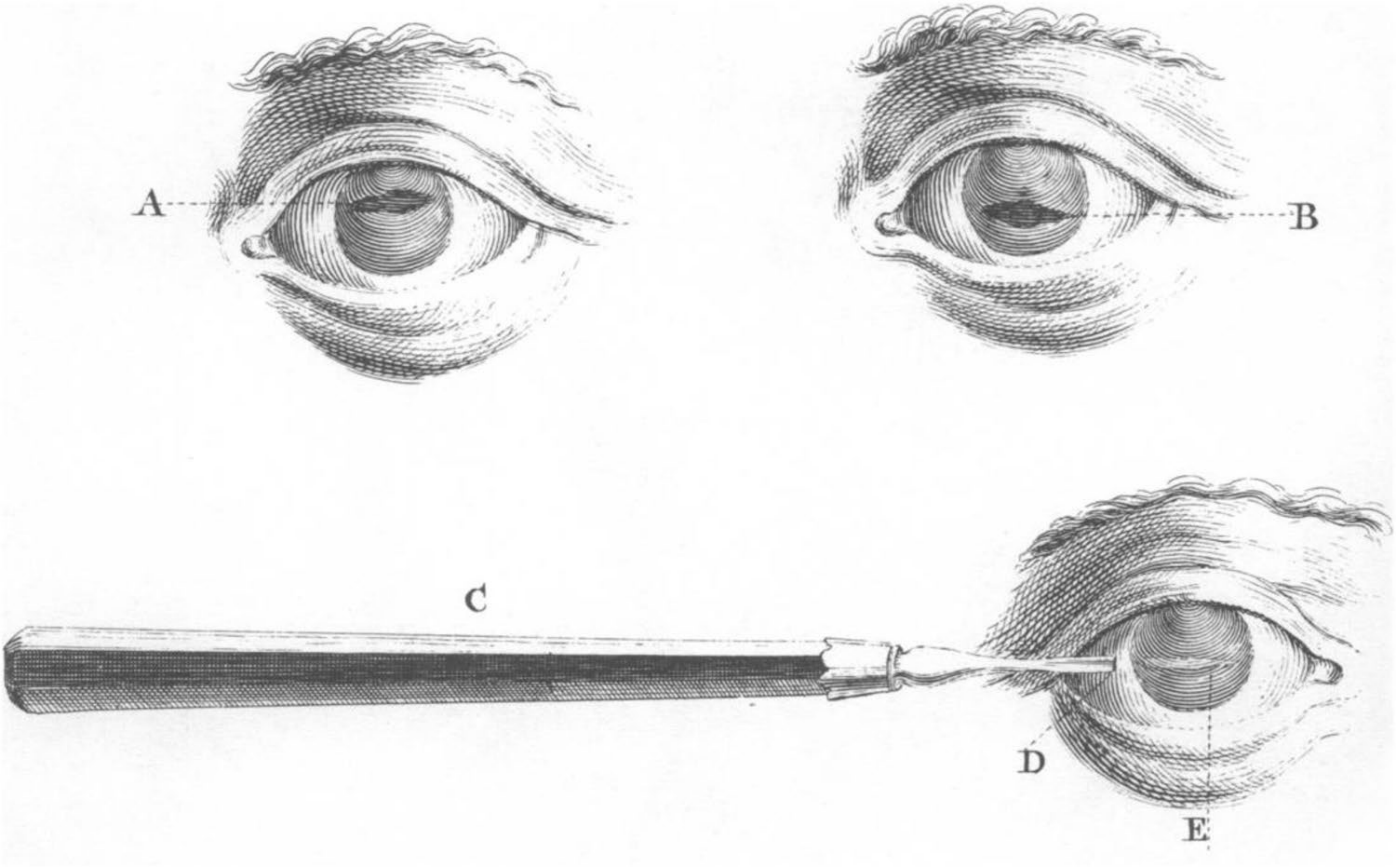
Coretomy or iridotomy technique of Chesselden (1727 CE) with the purpose of creating a new pupil in an iris with pupillary occlusion [101]

Despite the title of his book on glaucoma and iridectomy, Jaeger hardly wrote about iridectomy [42]. It seems that AvG discovered the benefits of iridectomy by chance. In his first article on coremorphosis, the word iridectomy was mentioned only once [99]. The idea behind coremorphosis was to make a hole in the iris in the event of a pupil, completely occluded by posterior synechiae. These adhesions were considered to be the main cause of recurrent iritis. Thus, one prevented recurrence and gave the patient an opening through which (s)he could see again. As already mentioned, AvG performed what he considered a risky coremorphosis in a monocular patient. While grabbing the iris with tweezers, a lot of yellow fluid came from behind it. The next morning, the iris was flat against the lens; a week later the patient's vision improved, and this continued for 4 years. AvG attributed this visual recovery to a reduction in inappropriate nutrients secreted by the choroid to the vitreous. In a footnote he added the incorrect assumption that with an occluded pupil the elevated IOP cannot reach the cornea [99]. Buoyed by this success, AvG performed more and more coremorphoses, even up to three times in one eye, and in another eye with complete fusion of the iris with the cornea. At the end, he added that he had no idea about the effect of coremorphosis on the IOP and that he had learned a lot from Louis Desmarres [100], who had written extensively about iridectomies [99]. Indeed, Desmarres had provided an overview of the development of coremorphosis. He started with Will Chesselden who called it a coretomy or iridotomy (Fig. 10) [101]. Scarpa described detachment of an iris part from the ciliary ligament as coredialysis or iridodialysis [97]. Baron Michael de Wenzel was the first to excise part of the iris, the corectomy or iridectomy [102]. Corencleisis or corectopy was performed by William Adams [103]. It was in fact an iridencleisis, but not under the conjunctiva to create a filter opening but in the cornea to prevent the new pupil from closing again. Desmarres himself used the expression “iris tearing [100].” Possibly due to advancing insight into the effect of his coremorphoses, AvG started his next article on iridectomy [104] refering to his previous one about iridectomy [99]; it actually was about coremorphosis and glaucoma was not mentioned in it. His first sentence started: “I can now add a new one to the healing effects of iridectomy, which I previously discussed in this archive……… I'm not saying anything new to most readers when I promote iridectomy as a remedy for the glaucomatous process [104].” He mentioned that antiphlogistics, diaphoretics, diuretics, laxative drugs, mercury and mydriatics all had no effect. Paracentesis only had a temporary IOP lowering result. AvG was very positive about the effect of iridectomy for the treatment of prodromal, acute inflammatory, and late inflammatory glaucoma. He claimed improvement of visual acuity and VF but not of the excavation in chronic glaucoma, finding no benefit at all in amaurosis with excavation. He wondered whether iridectomy would reduce IOP in healthy eyes and thought he could detect this in rabbit eyes by palpation [104]. AvG also wrote that an iridectomy caused sympathetic acute glaucoma in the fellow eye [105], but Arlt and Donders doubted that. When Donders and Haffmans introduced glaucoma simplex, which they considered the same as AvG’s amaurosis with excavation, more order appeared in the enormous diversity of glaucoma terminology [63]. However, doctors were not yet aware of the importance of open access to Schlemm’s canal in the ACA. A year-long struggle ensued between proponents of inflammation and those of nervous hypersecretion as a cause of glaucoma. Bowman introduced the iridectomy in England in 1857 and took up the cause for AvG against an anonymous article in the Dublin Journal of Medical Science, that called iridectomy “the glaucoma dodge [70].” Only after Knies’ articles [68], did doctors realize the importance of an open ACA and could they better distinguish ACG from POAG and secondary glaucoma.

## Where to go with glaucoma research?

We have seen that knowledge about all aspects of glaucoma increased over the centuries in fits and starts. Apparently, progress depended on someone who was more curious and stubborn than others, who often copied each other’s well-trodden paths. Why is it that 160 years after Donders and AvG, we still don’t know where POAG comes from? The servile offering of pleasing writing to superiors, ecclesiastical or secular, who suppressed free thought, slowly disappeared at the beginning of the nineteenth century. Antoine Demours dedicated his book to the king shortly after the French Revolution [106], but three years later to his colleagues at the Royal Academy [107]. Would female input have helped us sooner, given the total absence of female textbook authors until 1950? Mutual animosity and priority claims, regularly appearing in publications from Bartisch and Taylor, to Schlemm and Sichel are not conducive to collaboration. Does master-apprentice medical training distort young physicians? Physicists and chemists who work together in groups on a problem are often surprised by the hierarchical, cooperation-inhibiting, oppressive relationships in medical research and hospitals. I have been bombarded with advertising from the pharma industry for more than 50 years and cannot remember coming across any long-term glaucoma project sponsored by them. Numerous studies exist, whether an IOP-relieving drug works for a few months or years. But good studies on the long-term effect of POAG treatment after 10 to 15 years can be counted on one hand [108]. Are we teaching young researchers enough that good historical research can prevent duplication and disappointment before the start of a study? Did the authors of a 2000 CE article on VFL in wind instrument players [109] know that this had already been described in 1622 [110]? Do we not focus too much on obvious, less essential questions rather than e.g. on the possibility that glaucoma is primarily a neurological disorder due to mitochondrial, ribosomal, or different protein dysfunction? Are we publishing to advance the profession so we can treat people better or for our own well-being? Is writing just a conditio sine qua non for an academic career or is publishing just a business model for tycoons instead of, for example, selling food? Shouldn't reviewers and editors be more critical when mountains of articles about the latest hype pollute our journals and waste our time? Does publication pressure hinder collaboration, even at a national, let alone international level? And make large publishers not huge profits for their shareholders on the backs of young researchers, who must go to great lengths to secure a small grant? In doing so, are these companies not strangling the investigators they will need to survive in the future? We can't expect Gemini AI to solve the POAG riddle without researchers, can we? Wouldn't it be better for pharma and printing companies to create substantial grants for good, multidisciplinary collaborations on a topic like POAG? Why should this research only be paid by the universities and ultimately by the taxpayer?

POAG is a difficult condition to investigate clinically, due to its slow progression and relatively low prevalence compared to e.g. cardiovascular diseases. There have been dozens of hypothetical risk factors for POAG that have been stranded by insufficient evidence. They include for example loss of neuroprotection, cerebrospinal and intraocular fluid dynamics, gut microbiome changes and genetic or epigenetic changes. Of the population, 7% seem to suffer from chronic constipation, a risk factor for death from cardiovascular disease and cerebrovascular accidents [111], Could constipation be an unexamined risk factor for glaucoma, as Benedict noted long ago, possibly to side effects of opiates [38]? Many hypotheses cannot be confirmed due to small group sizes and possible interactions with other factors. The biggest mystery of POAG to me is after many years of research its heredity. We know that black ethnicity, and a positive family history are risk factors for glaucoma [112]. How is it possible that top geneticists in the GIST study with 2,000 well-documented persons with POAG and 3000 family members can barely find POAG genes [113]? The genes that have been found only seem to work in polygenic models. Could it be that POAG, like retinitis pigmentosa or amyotrophic lateral sclerosis [114], is one phenotype under which there are dozens of genotypes that we ophthalmologists cannot distinguish?

I have promised you an overview of glaucoma thinking over the last 2500 years. Table 1 lists many names given to what was considered glaucoma, which in the mid-19th century became increasingly fancy. Table 2 deals with the symptoms and Table 3 with the signs of and hypotheses about glaucoma.

**Table 1:** Various names for and associations with glaucoma through the ages

	(B)CE	1*	2	3	4	5	6	7	8	9	10	11	12	13	14	15	16	17	18	19	20	21	22
Hippocrates [14]	400		x																				
Aristotle [14]	350		x																				
Rufus [14, 15, 147]	65CE		x												x								
Galen [15]	200		x												x								
Oribasius [15]	370		x												x								
Aetius [115]	550		x												x								
Avicenna [116, 117]^⁑^	1000		x																				
Actuarius [118]	1300		x												x								
Chauliac [119]	1363	x													x								
Canamusali [120]	1499	x																					
Hali [121]	1499						x																
Arculanus [122]	1533	x	x																				
Allemannus [118]	1557		x																				
Nonnus [123]	1568		x																				
Bartisch [96]	1583	x													x								
Guillemeau [124]	1585		x																				
Banister [110]	1622	x	x																				
Plempius [125]	1632	x	x																				
Major [126]	1673	x	x																				
Bonet [19]	1700	x	x																				
Hovius [127]	1702	x	x																				
Maitre-Jan [128]	1707	x	x																				
Boerhaave [21, 149]^§^	1708	x	x												x								
Brisseau [34]	1709		x																				
Heister [129]	1713	x	x																				
Woolhouse [55]	1717	x	x																				
Palfyn [130]	1718	x																					
Pemberton [131]	1719		x																				
De Saint Yves [35]	1722	x	x																				
Morgagni [33]	1723	x	x																				
Taylor [54]	1736	x																					
Platner [132]	1745		x																				
Michaelis [133]	1753		x												x								
Porterfield [134]	1759	x																					
Gendron [41]	1770	x	x																				
De Wenzel [102]	1786	x	x																				
Bernard [118]	1794		x																				
Scarpa [97]	1801		x																				
Arrachart [135]	1805	x	x												x								
Desmonceaux [136]	1806	x	x																				
Autenrieth [137]	1808		x	x																			x
Benedict [69]	1809		x								x	x			x								
Beer [43]	1813		x												x						x		
Wardrop [23]	1818		x																				
Delarue [138]	1820	x	x												x								
Demours [106, 107]	1821		x																				
Guthrie [150]	1827		x												x								
Schön [139]	1828	x	x												x								
Fabini [140]	1831	x	x												x								
Jüngken [44]	1832	x	x												x								
Rosas [87]^‡^	1834		x	x																	x		
Schroeder [141]	1841		x																				
Sichel [47]	1841		x																			x	
Himly [142]	1843		x								x				x						x		
Warnatz [59]	1844		x				x	x					x		x								
Tavignot [39]	1846		x																				
Desmarres [100]	1847		x												x								
Deval [45, 151]	1851		x				x	x															
Mackenzie [98]	1853		x																				
Jaeger [27, 42]	1854		x				x	x													x		
von Graefe^†^			x	x	x	x	x	x			x			x		x	x	x		x	x		
Donders [24]	1855		x				x	x						x		x							
Arlt [143]	1856		x																				
Bowman [70]	1860		x				x	x		x	x												
Haffmans [6, 63]	1861		x								x			x		x							
Knies [67]	1876		x	x			x				x		x				x				x	x	
Weber [144]	1877		x				x	x	x		x	x				x	x		x		x	x	x
Greenway [95]	1880		x				x																
Manfredi [145]	1884		x														x				x		

^1*^Gutta serena as blindness or glaucoma

^2^Glaucoma any meaning

^3^Glaucomatous choroiditis, glaucoma chorioideum

^4^Posterior sclerochoroiditis

^5^Prodromal, imminent or incipient glaucoma

^6^(Sub) acute glaucoma

^7^Chronic glaucoma

^8^Malign glaucoma

^9^Fulminating glaucoma, florid glaucoma

^10^Inflammatory glaucoma, acute or chronic, glaucoma cum ophthalmia, inflammatory amaurosis

^11^Non-inflammatory glaucoma

^12^Absolute or amaurotic glaucoma

^13^Amaurosis or blindness with excavation

^14^Black or green cataract, cataracta glaucomatosa, hyaloidea or viridis

^15^Simple or primary glaucoma

^16^Secondary (acute) or traumatic glaucoma

^17^Sympathetic glaucoma

^18^Hyperopic glaucoma

^19^Hereditary glaucoma

^20^Angineurotic, arthritic, hemorrhagic, hypersecretion or retinal detachment glaucoma

^21^Nervous or apoplectic glaucoma

^22^Degenerated, phtysic or scabious glaucoma

^⁑^Only as colour, not as glaucoma

^§^Acroamatic publications by his students, mostly after his death

^‡^In addition, vitreous, choroidal and retinal glaucoma

^†^In his numerous articles over the years, von Graefe used many different expressions for glaucoma

**Table 2:** Glaucoma symptoms through the ages

	(B)CE	1*	2	3	4	5	6	7	8	9	10	11
Hippocrates [14]	400		x								x	
Aristotle [14]	350		x									
Ptolemy [14]	50CE										x	
Rufus [14, 15, 146, 147]	65		x									x
Galen [15]	200		x								x	
Chauliac [119]	1363	x	x	x		x	x					
Canamusali [120]	1499	x	x			x						
Hali [121]	1499		x									
Arculanus [122]	1533		x									
Bartisch [96]	1583	x	x	x		x						x
Guillemeau [124]	1585		x									
Banister [110]	1622	x	x									
Willis [147]	1676										x	
De la Hire [148]	1685										x	
Bonet [19]	1700	x	x									
Maitre-Jan [128]	1707	x	x									
Boerhaave [21, 149]^§^	1708	x	x			x					x	
Brisseau [34]	1709	x	x									
Heister [129]	1713	x	x			x						
Woolhouse [55]	1717	x	x									
Palfyn [130]	1718	x	x									
Pemberton [131]	1719		x									
De Saint Yves [35]	1722	x	x	x		x					x	
Morgagni [33]	1723	x	x	x								
Taylor [54]	1736	x	x			x						
Platner [132]	1745					x						
Gendron [41]	1770	x	x	x								
De Wenzel [102]	1786	x	x			x						
Scarpa [97]	1801		x			x						
Arrachart [135]	1805	x	x									
Desmonceaux [136]	1806	x	x									
Autenrieth [137]	1808		x	x								
Benedict [69]	1809		x			x						
Beer [43]	1813		x			x					x	
Wardrop [23]	1818	x	x									
Delarue [138]	1820	x	x									
Demours [106, 107]	1821	x	x	x		x	x					
Purkinje [90]	1825										x	
Guthrie [150]	1827	x	x	x		x						
Schön [139]	1828	x	x									
Fabini [140]	1831		x	x		x						
Jüngken [44]	1832	x	x			x						x
Rosas [87]	1834		x	x	x	x			x		x	x
Schroeder [141]	1841		x	x								
Sichel [47]	1841		x	x		x	x	x			x	x
Himly [142]	1843		x	x		x			x			
Warnatz [59]	1844		x	x		x		x	x		x	
Tavignot [39]	1846		x			x						x
Desmarres [100]	1847		x			x		x				
Deval [45, 151]	1862	x	x									
Mackenzie [98]	1853		x	x		x						
Jaeger [27, 42]	1854		x									
Von Graefe^†^			x	x	x	x	x	x		x	x	
Donders [24]	1855		x	x							x	
Arlt [143]	1856		x	x		x	x					
Bowman [70]	1860		x	x								
Haffmans [6, 63]	1861		x		x	x					x	
Knies [67]	1876		x	x		x		x			x	
Weber [144]	1877	x	x			x						
Terson [152]	1907											

^1*^Gutta serena, goutte sereine

^2^Visual loss

^3^Foggy vision

^4^Halos around lights

^5^Headache / eye pain

^6^Nausea, vomiting

^7^Occasional, discontinuous symptoms

^8^Periocular paresthesia

^9^Corneal anaesthesia

^10^Visual field loss, be it under another name, or scotoma

^11^Depression/ suicide

**Table 3:** Glaucoma signs through the ages

	(B)CE	1	2	3	4	5	6	7	8	9	10	11	12	13	14	15
Hippocrates [14]	400								x							
Aristotle [14]	350								x							
Rufus [14, 15, 147]	65CE								x	x				x		
Galen [15]	200								x	x						
Oribasius [15]	370									x						
Chauliac [119]	1363						x							x		
Canamusali [120]	1499		x													
Hali [121]	1499									x						
Arculanus [122]	1533										x			x		x
Alemannus [118]	1557										x					
Bartisch [96]	1583								x	x				x		
Guillemeau [124]	1585						x		x							
Banister [110]	1622						x		x	x					x	
Plempius [125]	1632								x		x					
Bonet [19]	1700		x						x	x						
Maitre-Jan [128]	1707						x							x		
Boerhaave [21, 149]^§^	1708	x	x	x						x						
Brisseau [34]	1709								x		x					
Heister [129]	1713		x	x			x		x	x	x	x				
Palfyn [130]	1718		x				x			x						
De Saint Yves [35]	1722		x				x		x	x	x			x		
Morgagni [33]	1723									x	x	x		x		x
Taylor [54]	1736	x					x		x						x	
Platner [132]	1745								x	x	x				x	
Michaelis [133]	1753						x									
Gendron [41]	1770						x		x					x		
De Wenzel [102]	1786								x					x		x
Scarpa [97]	1801						x			x	x			x	x	x
Arrachart [135]	1805										x	x			x	
Desmonceaux [136]	1806								x		x					
Autenrieth [137]	1808		x									x				x
Benedict [69]	1809	x					x		x	x	x				x	
Beer [43]	1813			x			x		x	x	x					x
Wardrop [23]	1818								x		x					x
Delarue [138]	1820						x				x					
Demours [107]	1821	x					x		x		x				x	
Weller [153]	1826														x	x
Guthrie [150]	1827	x	x	x			x			x	x					
Schön [139]	1828		x			x				x				x		
Mackenzie [98]	1830	x	x				x		x	x	x	x		x	x	x
Fabini [140]	1831	x	x	x			x			x	x				x	
Jüngken [44]	1832		x				x	x	x		x	x				x
Lawrence [154]	1833											x				x
Rosas [87]	1834	x	x	x	x		x	x	x	x	x	x			x	x
Weiss [155]	1837										x					
Schroeder [141]	1841	x							x			x			x	x
Sichel [47]	1841	x			x		x	x	x		x	x			x	x
Himly [142]	1843	x	x		x		x	x			x	x				x
Warnatz [59]	1844			x			x	x	x	x	x				x	
Tavignot [39]	1846	x	x	x	x	x	x	x	x	x						
Desmarres [100]	1847				x		x				x	x				
Deval [45, 151]	1851	x	x								x	x	x			
StellwagvC [156]	1853											x				
von Graefe^†^							x		x		x	x	x	x	x	
Jaeger [27, 42]	1854		x						x	x	x		x	x	x	
Donders [24]	1855	x	x				x						x		x	
Arlt [143]	1856	x	x				x		x	x	x	x				x
Bowman [70]	1860		x				x		x						x	x
Haffmans [6, 63]	1861														x	x
Knies [67]	1876	x		x		x	x	x	x				x	x		
Weber [144]	1877	x	x	x			x			x		x		x	x	x
Terson [152]	1907														x	

^1^Varicose conjunctival, scleral, iris vessels

^2^Inflammation

^3^Lacklustre / opaque cornea

^4^Corneal ulcers as end stage glaucoma

^5^Shallow or flat anterior chamber

^6^Sluggish or wide, fixed pupil

^7^Green-brown or lead coloured iris

^8^Green-bluish coloured lens

^9^Opaque lens

^10^Green or inflamed vitreous

^11^Damaged or inflamed choroid

^12^Pulsating central retinal artery

^13^Damaged or excavated optic disc/ nerve

^14^Elevated IOP

^15^Damaged or insensible retina

^§^Acroamatic publications by his students, mostly after his death

^†^In his numerous articles over the years, von Graefe used more different expressions for glaucoma such as secondary optic disc excavation

However, variable multi-causality is common and difficult to investigate. If several relevant factors, including genes, play a causal role in the development of POAG, their effect must first be investigated and measured separately under the same conditions, and next in combination. If we cannot solve complex problems with more than two related variables well, an observational, intuitive trial and error approach seems to be the best option. The 170th anniversary of our oldest ophthalmic journal seems like a good time to exponentially increase the glaucoma legacy of AvG and Donders through well-collaborative, transparent, multidisciplinary research groups of sufficient size.

## Footnotes

1 All English quotations of AvG's words have been translated by the author.
