# Correction: An eternal hunt for glaucoma

**DOI:** 10.1007/s00417-024-06581-z

**Published:** 2024-07-18

**Authors:** Paulus T. V. M. de Jong

**Affiliations:** 1 Department of Retinal Signal Processing, The Netherlands Institute of Neuroscience, Royal Academy of Arts and Sciences, Meibergdreef 47, 1105 Amsterdam, BA The Netherlands; 2 Department of Ophthalmology, Amsterdam University Medical Center, Amsterdam, The Netherlands

**Graefe’s Archive for Clinical and Experimental Ophthalmology (2024) 262:1955–1975**

10.1007/s00417-024-06441-w

The article “An eternal hunt for glaucoma”, written by Paulus T. V. M. de Jong, was originally published Online First without open access. After publication in volume 262, issue 7 page 1955–1975, the author decided to opt for Open Choice and to make the article an open access publication. Therefore, the copyright of the article has been changed to © The Author(s) 2024 and the article is forthwith distributed under the terms of the Creative Commons Attribution 4.0 International License (http://creativecommons.org/licenses/by/4.0/), which permits use, duplication, adaptation, distribution and reproduction in any medium or format, as long as you give appropriate credit to the original author(s) and the source, provide a link to the Creative Commons license, and indicate if changes were made. The images or other third party material in this article are included in the article’s Creative Commons licence, unless indicated otherwise in a credit line to the material. If material is not included in the article’s Creative Commons licence and your intended use is not permitted by statutory regulation or exceeds the permitted use, you will need to obtain permission directly from the copyright holder. To view a copy of this licence, visit http://creativecommons.org/licenses/by/4.0.

The original article was corrected.

